# Pseudo-Appendicitis in an Adolescent With COVID-19

**DOI:** 10.7759/cureus.9394

**Published:** 2020-07-25

**Authors:** Kulachanya Suwanwongse, Nehad Shabarek

**Affiliations:** 1 Internal Medicine, Lincoln Medical Center, New York City, USA

**Keywords:** appendicitis, covid-19, acute abdomen, sars-cov-2, surgical abdomen

## Abstract

Coronavirus disease 2019 (COVID-19) pandemic is a global health emergency in 2020. Patients with COVID-19 may present with variable clinical features, involving pulmonary, gastrointestinal, neurological, and cardiovascular symptoms. Notwithstanding, the acute abdomen as a presentation of COVID-19 is rare. We report an adolescent with confirmed COVID-19, initially presented with acute abdominal pain mimicking appendicitis. Our case highlights the inaccuracy of using clinical diagnosis for surgical abdomen in the COVID-19 era. Clinicians should perform screening COVID-19 tests in patients presenting with acute abdominal pain before admitting the patients to implement proper preventive measures in order to reduce viral transmission to other patients and healthcare professionals. Confirmed COVID-19 patients with acute abdomen may need proper imaging tests before surgery to avoid iatrogenic complications.

## Introduction

Coronavirus disease 2019 (COVID-19), first reported in Wuhan, China, in early December 2019, has now become an international pandemic. The causative agent, severe acute respiratory syndrome coronavirus 2 (SARS-CoV-2), binds angiotensin‐converting enzyme 2 (ACE 2) receptors to enter human cells resulting in spectrums of diseases, ranging from flu-like illnesses to fatal pneumonia [1]. Besides, SARS-CoV-2 may cause unpredictable complications in various organ systems. A recent meta-analysis, which contained 17 studies and 2,477 patients, found that 13% of COVID-19 patients had gastrointestinal (GI) symptoms; anorexia was the most prevalent followed by diarrhea and nausea/vomiting [2]. Nonetheless, acute abdomen as a presentation of COVID-19 is rare, and its correlation to COVID-19 features and prognosis remains undetermined. Herein, we present a COVID-19 patient who initially presented with pseudo-appendicitis.

## Case presentation

An 18-year-old female patient presented to the emergency department due to worsening abdominal pain. She reported the pain began in the periumbilical area three days prior but later shifted to the right iliac fossa six hours before. Pain score was 8 out of 10, and movement aggravated the pain. She had anorexia, nausea, vomiting, and some watery diarrhea but denied fever and respiratory symptoms. She did not have previous medical problems and was not taking medications except acetaminophen for her pain. Initial vital signs were unremarkable, including normal temperature at 36.8 degrees Celsius. Lung and heart exams were normal. Examination of the abdomen revealed right lower quadrant tenderness with voluntary guarding and positive rebound.

Her blood test results are shown in Table 1. She had neutrophilia, lymphopenia, and elevated erythrocyte sedimentation rate (ESR) and C-reactive protein (CRP). D-dimer and ferritin were normal.

**Table 1:** The patient's blood results WBC, white blood cell count; ESR, erythrocyte sedimentation rate; CRP, C-reactive protein; CPK, creatine phosphokinase

Blood parameter	Patient’s value	Normal values	Unit
WBC	8,050	4,080-10,800	cells/mcl
Neutrophils	80	44-77	%
Lymphocytes	15	20-45	%
ESR	63	0-15	mm/hr
CRP	1.21	<0.40	mg/dl
Procalcitonin	0.06	<0.08	ng/ml
Lactate	1.2	<2.2	mmol/L
CPK	95	<120	U/L
Troponin T	<0.01	<0.01	ng/ml
D-dimer	177	<230	ng/ml
Ferritin	9	<150	ng/ml

**Figure 1:**
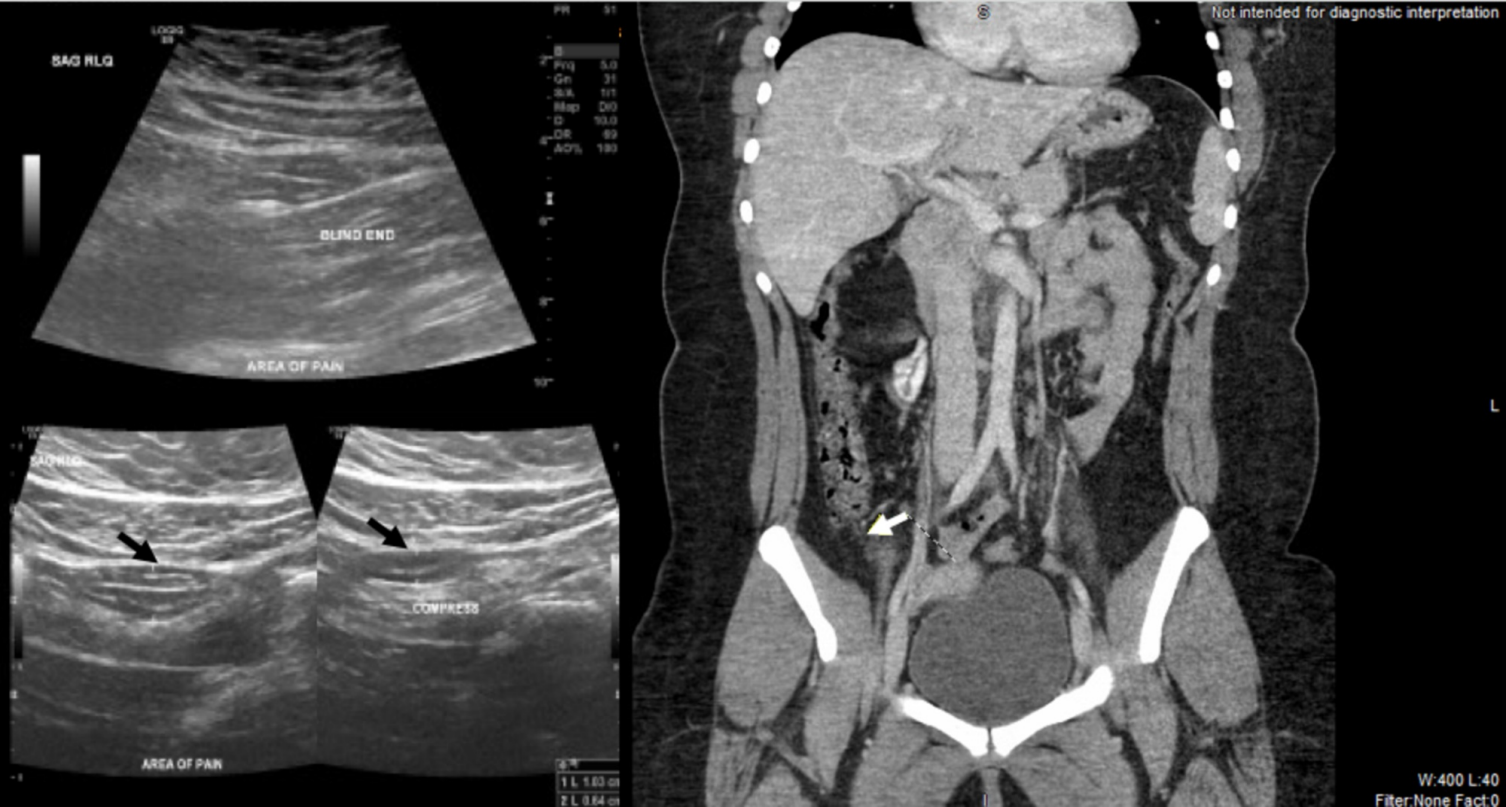
Ultrasound (left) shows non-compressible appendix (black arrow) and CT (right) shows normal appendix (white arrow)

Pregnancy test was negative, and urine analysis was unremarkable. Her chest x-ray (CXR) was normal. Pelvic ultrasound (USG) illustrated a non-compressible mildly dilated appendix with a maximum diameter of 10 mm, which was consistent with possible appendicitis; the adnexal structures appeared normal (Figure 1, left). CT of the abdomen was normal with no appendicitis and absence of lung bases infiltration (Figure 1, right). Universal SARS-CoV-2 reverse transcription polymerase chain reaction (RT-PCR) was sent despite the absence of suggestive COVID-19 symptoms but returned positive. The patient was kept under observation, with frequent reassessment. Her pain disappeared the next day; she tolerated oral intake and was discharged home. She did not receive specific COVID-19 treatment and never developed respiratory symptoms or fever. The patient did not have acute appendicitis for up to two months following discharge.

## Discussion

Acute abdominal pain in COVID-19 patients poses a diagnostic dilemma to clinicians. Delaying management of the surgical abdomen can result in grave complications and worsen mortality. In contrast, performing unnecessary surgery in COVID-19 patients causes iatrogenic morbidity and mortality, more strain on healthcare resources, and high-risk exposure for healthcare workers involved in operative fields.

Saeed et al. revealed that two out of nine (22%) COVID-19 patients, who presented with an acute abdomen without respiratory symptoms, had surgical abdominal diseases (one appendicitis and one cholecystitis) [3]. Two smaller case series, each contained three COVID-19 patients with acute abdominal pain, found none (0%) had surgical conditions [4,5]. The actual incidence of surgical abdomen in COVID-19 patients demands larger studies.

Our case report highlights the limited usefulness of clinical diagnosis for the surgical abdomen in COVID-19 patients. Fever, anorexia, nausea, vomiting, and abdominal pain are overlapping symptoms in these two conditions. SARS-CoV-2 causes lymphocytopenia and neutrophilia mimicking 'left shift' in the surgical abdomen. Our patient had clinical syndromes resembling appendicitis with the Alvarado score of 7 (1 for shifting pain, anorexia, nausea, right iliac fossa tenderness, a shift of white blood cell count to the left, and 2 for rebound pain). Elevated inflammatory markers further support the diagnosis of appendicitis in our patient. The patient's USG finding suspected appendicitis, but the CT scan was normal. Her pain disappeared following symptomatic management.

This case highlights the need for CT diagnoses of appendicitis. We suggest clinicians performing universal SARS-CoV-2 tests in patients presenting with an acute abdomen. COVID-19 patients with severe abdominal pain should also have proper imaging before surgery unless they have contradictions in order to avoid harmful surgery. 

**Figure 2:**
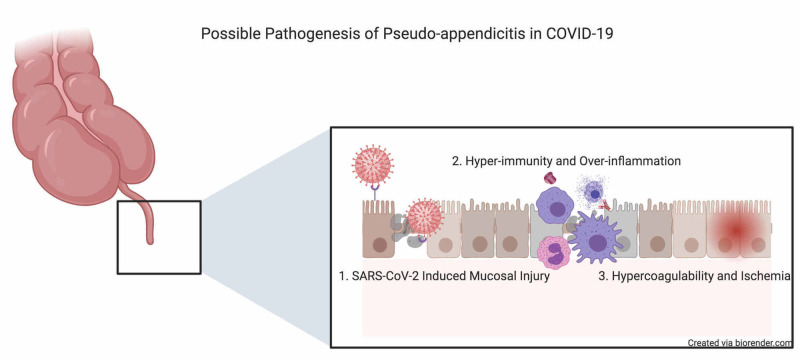
Proposed mechanisms of COVID-19 induced pseudo-appendicitis SARS-CoV-2, severe acute respiratory syndrome coronavirus 2

The mechanisms underlying the acute abdomen in COVID-19 have not yet been elucidated. Saeed et al. proposed that SARS-CoV-2 binds ACE-2 receptors in GI tract and invades the GI mucosa causing pain [3]. We agree with this hypothesis, but suggest that hyper-inflammation and dysregulation of immune cells may also play a role. Besides, prothrombotic states in COVID-19 patients can lead to organ ischemia and inflammation, mimicking surgical abdominal diseases. In addition, pseudo-appendicitis in patients with an infectious disease may occur due to acute mesenteric lymphadenitis, which can be accurately diagnosis by CT scan. Figure 2 summarizes the possible pathogenesis of pseudo-appendicitis in COVID-19 patients. Notwithstanding, the acute abdominal pain in our patient may not relate to COVID-19 and the occurrence of both conditions was only coincidence. More research is needed to verify our hypothesis. 

## Conclusions

COVID-19 patients can present with an acute abdomen with or without underlying surgical diseases. Clinical symptoms, signs, and laboratory values of COVID-19 and surgical abdominal conditions overlap, challenging physicians’ abilities to make accurate diagnosis and management to avoid iatrogenic complications. Until the pandemic resolved, SARS-CoV-2 tests should be performed in all patients with acute abdominal pain who will be admitted to inpatient care to provide proper protective isolation measures in order to prevent nosocomial viral transmission. 
